# Medial patellofemoral ligament reconstruction using nonresorbable sutures yields comparable outcomes to reconstruction with a pedicled quadriceps tendon autograft when performed in addition to bony risk factor correction

**DOI:** 10.1007/s00167-022-07104-1

**Published:** 2022-08-16

**Authors:** Danko Dan Milinkovic, Felix Zimmermann, Peter Balcarek

**Affiliations:** 1 Center for Musculoskeletal Surgery, Charité-University Medicine Berlin, Luisenstrasse 64, 10117 Berlin, Germany; 2 Berufsgenossenschaftliche Unfallklinik Ludwigshafen, Ludwigshafen am Rhein, Germany; 3 Arcus Sportklinik, Pforzheim, Germany; 4 Department of Trauma Surgery, Orthopaedics, and Plastic Surgery, University Medicine Göttingen, Göttingen, Germany

**Keywords:** MPFL reconstruction, Synthetic graft, Autograft, Risk factors, Bony correction, BPII 2.0

## Abstract

**Purpose:**

To evaluate the results for reconstruction of the medial patellofemoral ligament using synthetic nonresorbable sutures (S-MPFL-R) in comparison to MPFL-R using quadriceps tendon autograft (QT-MPFL-R) in patients undergoing simultaneous correction of anatomic risk factors for lateral patellar instability (LPI) at a minimum of 2 years of follow-up.

**Methods:**

Between November 2018 and June 2019, 19 patients (male/female 8/11; mean age 26 ± 7 years) underwent S-MPFL-R (FiberTape^®^) in combination with the correction of predisposing risk factors for LPI. The control group of 38 patients (male/female 16/22, mean age 26 ± 6 years) who underwent QT-MPFL-R was matched 1:2 by sex, age, anatomic risk factors, and concomitant surgical correction of bony risk factors. The Banff Patella Instability Instrument 2.0 (BPII 2.0) and a numerical analog scale (NAS 0–10) for patellofemoral pain and subjective knee joint function were used to assess patients’ reported quality of life before and after surgery.

**Results:**

The BPII 2.0 score increased from 35.0 ± 21.7 points to 79.7 ± 13.3 points (*p* < 0.0001) in the S-MPRL-R group and from 44.3 ± 19.6 points to 80.9 ± 15 points (*p* < 0.0001) in the QT-MPFL-R group from preoperatively to postoperatively, respectively, without any significant difference between the groups. In the S-MPFL-R group and QT-MPFL-R group, 95% (18/19) and 92% (35/38) of patients, respectively, crossed the minimally clinically important difference reported for the BPII 2.0. NAS values for pain and subjective knee joint function improved significantly in both groups (*p* < 0.0001, *p* < 0.0001) without any significant difference between the groups at the final follow-up.

**Conclusions:**

This study demonstrates that nonresorbable sutures can serve as a viable option for MPFL-R, yielding comparable outcomes compared to quadriceps tendon autograft reconstruction when performed concomitantly with the correction of anatomic risk factors for LPI. This option reduces the need for autologous tendon harvesting or the use of allografts for MPFL-R.

**Level of evidence:**

Level III.

## Introduction

Reconstruction of the medial patellofemoral ligament (MPFL-R) is one of the most common procedures for the surgical treatment of recurrent patellar instability [1, 16, 24]. In recent years, numerous operative techniques, graft options, and fixation methods have been described, showing that with the more widespread utilization of MPFL-R surgery, complications and failure rates are decreasing [1, 12, 25, 44]. Although not entirely consistent within the literature, more frequent implementation of concomitant procedures, aiming to correct bony pathoanatomy in addition to MPFL-R, has been shown to reduce the rate of patellar redislocation, while improving clinical and patient-reported outcomes [4, 13, 49].

The majority of currently available techniques require harvesting of an autologous tendon graft (i.e., quadriceps, gracilis or semitendinosus tendon), which can be technically challenging and potentially lead to donor-site morbidities and functional deficits [17, 24, 30, 33, 41]. Therefore, MPFL-R techniques with the use of synthetic materials have gained increased interest in recent years [8, 15, 20, 21, 23, 29]. However, reports on the efficiency of these materials in MPFL-R surgery have thus far been scarce and, for the most part, published in the form of technical notes, case series and very few biomechanical evaluations [29, 32, 35, 36, 46].

The purpose of this study was to evaluate the results of a novel MPFL-R technique using synthetic nonresorbable sutures in comparison to MPFL-R using a quadriceps tendon autograft in patients undergoing simultaneous correction of anatomic risk factors for lateral patellar instability (LPI) at a minimum of 2 years of follow-up. The hypothesis was that the results of synthetic MPFL-R are not inferior to those of tendon autograft reconstruction.

## Materials and methods

This study represents a retrospective analysis of prospectively collected data that has been approved by the local ethics committee (Medical Council Baden-Württemberg F-2019–070). Between November 2018 and June 2019, the first 19 patients (male/female 8/11; mean age 26 ± 7 years) who underwent MPFL-R using nonresorbable sutures (S-MPFL-R) (FiberTape^®^, Arthrex Co., Nepales, Florida, USA) in combination with the concomitant correction of predisposing pathoanatomic factors of patellar instability comprised the study group for this investigation. Thirty-eight patients (male/female 16/22, mean age 26 ± 6 years) who underwent MPFL-R with a pedicled quadriceps tendon autograft (OT-MPFL-R) comprised the control group. For the best possible homogeneity between the groups, QT-MPFL-R patients were selected out of a total of 140 MPFL-Rs (years 2017–2019) by matching them 2:1 to the S-MPFL-R group regarding sex, age, anatomic risk factors, and their concomitant bony correction during MPFL-R (see Tables 1 and 2).

**Table 1:** Demographics and pathoanatomic risk factor profile for both patient cohorts

Factors	S-MPFL-R *n* = 19	QT-MPFL-R *n* = 38	*p* value
Male/female	6/13	12/26	n.s
Age (years)	26.2 ± 7.0 (14–35)	26.6 ± 6.0 (15–39)	n.s
Follow-up (months)	24.6 ± 1.3 (24–29)	35.8 ± 9.9 (24–57)	** < 0.001**
Trochlear dysplasia absent/mild/severe	1/5/13	0/8/30	n.s
Caton–Deschamps index	1.2 ± 0.3 (0.8–1.6)	1.2 ± 0.3 (0.7–1.7)	n.s
TT–TG distance (mm)	15.8 ± 5.0 (9–30)	15.6 ± 4.6 (7–26)	n.s
TT–PCL distance (mm)	23.0 ± 3.5 (13–28)	22.9 ± 3.2 (15–30)	n.s
Frontal plane deviation (°)^a^	− 0.7 ± 3.0 (− 8–3.4)	− 0.6 ± 3.1 (− 7–4.6)	n.s

^*^S-MPFL-R medial patellofemoral reconstruction with the synthetic graft, *QT-MPFL-R* medial patellofemoral ligament reconstruction with the quadriceps tendon graft, *TT–TG* tibial tuberosity–trochlear grove distance, *TT–PCL* tibial tuberosity–posterior cruciate ligament distance, *n.s.* non-significant

^a^Negative values indicate valgus deviation

**Table 2:** Overview of the concomitant procedures performed in addition to MPFL-R in both patients cohorts

Operative procedures	S-MPFL-R	QT-MPFL-R
MPFL-R + TTO	8	16
MPFL-R + TP	3	6
MPFL-R + TTO + TP	2	4
MPFL-R + DFO	6	12

^*^S-MPFL-R medial patellofemoral reconstruction with the synthetic graft, *QT-MPFL-R* medial patellofemoral ligament reconstruction with the pedicle quadriceps tendon graft, *MPFL-R* medial patellofemoral ligament reconstruction, *TTO* tibial tuberosity osteotomy, *TP* trochleoplasty, *DFO* distal femoral osteotomy

The validated Banff Patella Instability Instrument 2.0 (BPII 2.0) [7] was used to evaluate the patient-reported quality of life (QOL) prior to and at a minimum of 24 months after surgery. Additionally, a numerical analog scale (NAS 0–10) for the assessment of patellofemoral pain and subjective knee joint function was collected at the time of initial examination and at the final follow-up as previously published [49].

The inclusion criteria were as follows: (1) a history of recurrent LPI, defined as ≥ 2 patellar dislocations following a failed conservative treatment over a period of a minimum of 6 months; (2) clinically and radiologically diagnosed presence of at least one predisposing risk factor for LPI (please see below). The exclusion criteria were as follows: (1) previous MPFL-R and medial soft-tissue-stabilizing procedures, previous tibial tubercle osteotomy (TTO) and/or other bony procedures at the distal femur or proximal tibia (including osteotomy and trochleoplasty); (2) patellofemoral pain without objective findings of lateral patellar instability; and (3) previous knee ligament surgical procedures.

The evaluation of the parameters was determined by reaching a mutual consensus among the authors of the study. Routine radiographs, including standing long leg axis and true-lateral view of the knee joint (≤ 3 mm of overlap between the femoral condyles) and magnetic resonance imaging (MRI) scans were obtained for all patients in both groups. The patients were also screened for femoral and tibial torsional deformities, and torsional MRI scans were performed in cases with a clinically important rotational malalignment as previously published [3]. Data were evaluated for the presence of pathoanatomic abnormalities using predetermined thresholds and grading scales [49]: the severity of trochlear dysplasia [absent, low grade (Dejour type A), or high grade (Dejour types B–D)] [13]; patellar height, with a Caton-Deschamps index ≥ 1.3 recorded as elevated [11]; tibial tuberosity–trochlear groove (TT–TG) distance, with ≥ 16 mm considered elevated [5]; tibial tuberosity–posterior cruciate ligament (TT–PCL) distance, with ≥ 24 mm recorded as elevated [39]; ≥ 4° valgus malalignment [19] and > 25° of femur antetorsion recorded as elevated [3].

Accordingly, concomitant trochleoplasty was indicated in patients with Dejour type B or D trochlear dysplasia and the presence of a high-grade (grades II–III) J-sign; concomitant TTO was considered when the TT–TG distance exceeded 16 mm, the TT–PCL distance exceeded 24 mm, and/or when the Caton–Deschamps index was ≥ 1.3. Femoral derotation osteotomy or valgus correction osteotomy was considered when femoral antetorsion exceeded 25° and the valgus deformity was ≥ 4°, respectively. All surgical procedures were performed by the senior author of this study.

### Surgical technique

Until 2018, the QT-MPFL-R technique according to Fink et al. [17] was the standard technique consistently used for MPFL-R in our clinic [49]. Based on the favorable outcomes of the MPFL-R procedure with synthetic materials published by Lee et al. [23], we developed and began utilizing the FiberTape^®^ technique with soft-tissue patellar fixation in 2018. Primarily, this technique of stabilization was used in cases of primary LPI who had to have undergone refixation of an osteochondral flake fracture to decrease the additional intraoperative trauma. Since the results were favorable, this technique was implemented in cases of recurrent LPI with concomitant osseous procedures using the following technical steps:

The patient was placed in the supine position, with the operated leg fixed in the electric leg holder and a pneumatic tourniquet placed on the mid-portion of the thigh. A clinical examination under anesthesia and diagnostic arthroscopy using standardized anteromedial and anterolateral portals were performed in every patient.

**Fig. 1:**
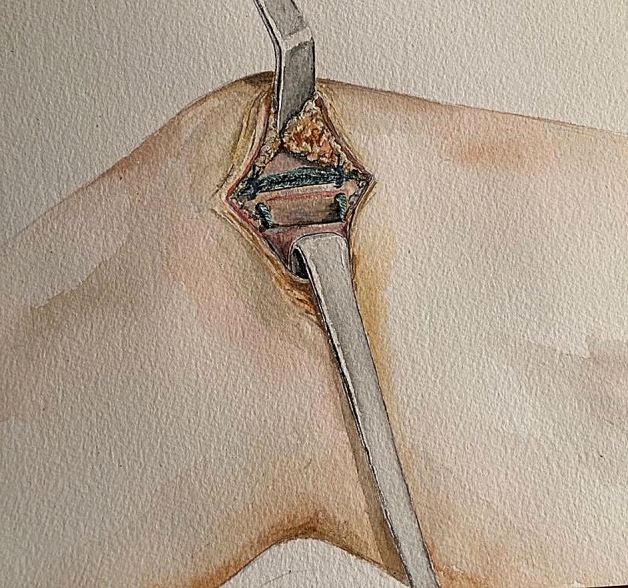
MPFL-R using nonresorbable suture tape with soft-tissue patellar fixation. The first and second layers of the medial retinaculum are opened approximately 1 cm from the medial patellar margin. The suture material (FiberTape^®^, Arthex Co., North Naples, Florida, USA) is passed through the proximal and distal origin of the native MPFL, and two topstitching seams (No. 2 Vicryl, Ethicon, Sommerville, NJ, USA) are added to the proximal and distal edges of the synthetic graft. Thereafter, the graft is guided between the second and the third layers of the medial retinaculum to the femoral tunnel

For S-MPFL-R, a medial parapatellar skin incision was made on the proximal 2/3 of the medial patellar margin, approx. 3–4 cm in length. Blunt dissection through the subcutaneous tissue allows for direct visualization of the medial patellar margin and medial retinaculum. The first and second layers of the medial retinaculum are opened approximately 1 cm from the medial patellar margin over a length of 2–3 cm, maintaining an intact joint capsule (third layer) [48]. A free needle was attached to one of the free ends of the nonresorbable suture material (FiberTape^®^, Arthex Co., North Naples, Florida, USA) and passed through the medial retinaculum at the proximal and distal ends of the native MPFL origin. Adding two topstitching seams (No. 2 Vicryl, Ethicon, Sommerville, NJ, USA) directly to the proximal and distal edges of the FiberTape® provides additional stability to the triangular construct (Fig. 1). Femoral tunnel positioning was determined under a true-lateral fluoroscopic view using predefined reference points according to Schöttle et al. [38] The femoral tunnel of approx. 40–50 mm in depth was created using a 5 mm cannulated drill bit. A suture lasso was passed through the tunnel from medial to lateral, allowing for later insertion of the synthetic graft. The free ends of the synthetic graft were then guided through the soft-tissue canal between the 2nd and 3rd layers of the medial retinaculum and inserted into the femoral tunnel*.* Afterward, the knee was moved through several full ROMs ending at approximately 60° of knee joint flexion. After applying mild tension of approximately 2 N, the graft is temporarily fixed on the lateral side of the thigh using an Overhold clamp. Then, the knee is extended, and the patella is checked for stability in extension, allowing two quadrants of mediolateral translation. Once adequate stability is achieved, the knee joint is again flexed to 60°. In this position, the final fixation is performed by inserting an interference screw (6 × 23 mm; Arthrex Co., North Naples, Florida, USA) into the femoral tunnel. The retinaculum was closed using additional single sutures (No 2–0 Vicryl, Ethicon, Sommerville, NJ, USA).

#### Postoperative treatment

The same postoperative rehabilitation protocol was used in both groups, with partial weight bearing for 3–4 weeks, followed by a gradual increase to full-weight bearing in the 5–6 weeks postoperatively as tolerated. Mobilization was initiated on the first day after the operation with active and passive exercises, including continuous passive motion (CPM) as tolerated. The patients underwent physiotherapy rehabilitation for a total of three months postoperatively. No bracing was applied in either of the groups. Jogging and mild sports activities were permitted at 12–16 weeks, and patients returned to sports activity at 5–6 months postoperatively. Patients underwent clinical and radiological evaluations at 6 and 12 weeks postoperatively.

### Statistical analysis

Continuous data were assessed for normality and are presented as the mean ± standard deviation (range). Categorical and dichotomous data are presented as frequency tabulations. Unpaired and paired two-tailed *t* tests, Fisher’s exact test, and the Mann–Whitney *U* test were used to assess differences between the pre- and postoperative clinical data and between the S-MPFL-R and the QT-MPFL-R groups. All analyses were performed using GraphPad Prism (version 4; GraphPad Software, San Diego, CA, USA). The level of significance was set at 0.05. The post hoc power analysis included all patients and was performed with G*Power-1 (version 3.1.3), yielding a power = 0.84 (effect size dz = 0.4, α error probability = 0.05) to detect a difference in postoperative BPII 2.0 score values between the groups.

## Results

The BPII 2.0 increased from 35.0 ± 21.7 points to 79.7 ± 13.3 points (*p* < 0.0001) in the S-MPRL-R group and from 44.3 ± 19.6 points to 80.9 ± 15 points (*p* < 0.0001) in the QT-MPFL-R group from preoperatively to postoperatively, respectively, without any significant difference between the groups (Table 3). In the S-MPFL-R group and QT-MPFL-R group, 95% (18/19) and 92% (35/38) of patients, respectively, crossed the minimally clinically important difference (MCID) reported for BPII 2.0 (MCID > 6.2 points) (n.s.) [22].

**Table 3:** Preoperative and postoperative score values for both patient cohorts

	S-MPFL-R	QT-MPFL-R	*p* value
BPII 2.0			n.s
Preoperative	35.9 ± 21.7	44.3 ± 19.6	
Postoperative	79.7 ± 13.3	80.9 ± 15.1	
* p* value	** < 0.0001**	** < 0.0001**	
NAS function			n.s
Preoperative	3.1 ± 2.5	4.2 ± 2.5	
Postoperative	8.1 ± 1.2	8.4 ± 1.4	
* p* value	** < 0.0001**	** < 0.0001**	
NAS pain			n.s
Preoperative	6.8 ± 2.1	5.9 ± 2.3	
Postoperative	2.1 ± 1.4	2.8 ± 3.5	
* p* value	** < 0.0001**	** < 0.0001**	

^*^BPII 2.0 Banff Patella Instability Instrument score, *NAS* numerical analog scale, *n.s.* non-significant

Patellofemoral pain and subjective knee joint function improved significantly in both groups (*p* < 0.0001, *p* < 0.0001), without any significant difference between them at the final follow-up (Table 3). None of the patients experienced failure of the MPFL-R with recurrence of patellar instability or subjective patellar subluxations. Evaluations were performed postoperatively at a mean of 24.6 ± 1.3 months (range, 24–29 months) (S-MPFL-R) and 35.8 ± 9.9 months (range, 24–57 months) (QT-MPFL-R) (*p* < 0.001).

No significant complications were reported in either of the two examined cohorts. In particular, in the S-MPFL-R group, there was no evidence of incompatibility or foreign body reaction to the synthetic material used. There were 3 patients in the study group and 3 in the control group who were treated with a concomitant tibial tubercle osteotomy, 2 in the study group and 3 in the control group who underwent valgus correcting osteotomy, and 3 in the study group and 2 in the control group who underwent derotational osteotomy and needed hardware removal at 10–12 months postoperatively.

## Discussion

The main findings of this study support the hypothesis that MPFL-R using nonresorbable sutures presents an effective and safe alternative to QT-MPFL-R, yielding satisfactory results in terms of patient-reported outcome measures and patellar stability when performed concomitantly with the correction of anatomic risk factors.

Surgical reconstruction of the MPFL is considered a cornerstone of operative treatment in LPI [1, 16, 24, 25]. Many techniques with various graft options and fixation methods have been successfully implemented in clinical practice, and the reported results have been favorable [16, 18, 26, 37, 44]. However, a variety of complications following MPFL-R have also consistently been reported, with anatomical femoral tunnel positioning and the correction of major pathoanatomy outlined as crucial for attaining adequate graft isometry and reducing the rates of patellar redislocation while increasing patellofemoral stability and functionality [4, 10, 31, 33, 34, 40, 41, 49].

The utilized graft (semitendinosus/gracilis, quadriceps, adductor magnus, or patellar tendon) does not appear to be a decisive factor for the outcomes [37]. In addition, there also seems to be a non-significant difference in terms of the redislocation rates and clinical outcomes depending on the graft fixation methods (drill holes, soft tissue, anchor, screw, etc.) [37]. Although complications related to autologous tendon harvesting have thus far only been mentioned as potential insults in studies reporting on the outcomes of autologous MPFL-R techniques [33, 41], donor-site morbidity has been consistently reported as a possible complication in ACL reconstruction using autologous tendons, including lesions to the *N. saphenous*, donor-site tenderness, hematoma, extensor lag and, in rare cases, muscle retraction and short-term loss of muscle function [9, 27, 42, 43]. The use of allografts for MPFL-R eliminates this potential donor-site morbidity but has limited availability in several countries.

The use of synthetic materials in ligament reconstructive surgery has gained increasing interest over the last few decades, but they have primarily been reserved for acute ligament repair and joint stabilization procedures [2, 6, 47]. Artificial grafts were utilized for MPFL-R, with initial reports dating back to 2000 [31]. Since then, there have been very few studies on this subject, with most of the available papers published in the form of technical notes, case reports, and small patient series [8, 15, 20, 21, 23, 36]. Although the techniques prove to be incoherent in regard to their main technical aspects, the use of various artificial grafts and different fixation methods, the most recently reported outcomes have been favorable, demonstrating that these materials could be utilized as safe and efficient alternatives to autograft options [8, 21, 23, 45].

One previous study compared the results of MPFL-R using FiberTape® sutures with gracilis tendon autograft reconstruction [23]. Using various outcome scores (Kujala score, Bartlett score, Tegner activity rating scale, SF-12 score, and Lysholm score), both procedures led to significant improvements after 48 months of follow-up, leading to the conclusion that the FiberTape® technique can yield comparable results to autograft reconstruction when performed in patients without major anatomical risk factors for LPI [23]. In their study, the presence of bony anomalies was considered contraindicative. Conversely, this study evaluated S-MPFL-R primarily as an adjunct to the correction of major anatomical risk factors for LPI, and the presented findings indicate that the sutures used can serve as a viable option for MPFL-R when performed concomitantly with these procedures as well.

Regarding the structural properties, biomechanical studies could demonstrate higher ultimate loads of synthetic materials with anchor fixation when compared to MPFL-R using gracilis tendon autografts [29, 35, 46]. Thushima et al. [46] compared the biomechanical properties of MPFL-R using FiberTape^®^ and knotless anchors versus semitendinosus autograft MPFL-R and soft anchor fixation and reported a significantly higher ultimate load to failure of the synthetic materials. The reported stiffness of the S-MPFL-R might raise certain concerns regarding the potential increase in the patellofemoral (PF) joint peak pressure. In this regard, Suganuma et al. [45] investigated the results of synthetic MPFL-R with arthroscopic control of patellofemoral congruence. Although no difference in knee function was found between groups, subjective evaluations were better in knee joints in which the patellae were positioned slightly lateral to the center of the trochlea groove than in those in which the patella was reduced to the strict center. In a biomechanical cadaver model, Mehl et al. [29] reported that repair of the MPFL with suture tape augmentation resulted in similar primary contact pressures and joint kinematics as reconstruction with a tendon graft. Nevertheless, exact positioning of the femoral tunnel and careful assessment of graft tensioning prior to its final fixation are essential steps in avoiding an overconstraint construct with consecutive PF-joint overload. However, evaluation of the specific biomechanical properties of this construct was beyond the scope of this study and is reserved for future investigations.

Recommendations regarding the optimal knee joint flexion angle for MPFL-graft fixation range from 20° to 90° of knee joint flexion [14, 28, 34, 40]. Sakamoto et al. [35] investigated the effect of the knee flexion angle during graft fixation on PF-joint contact pressure in MPFL-R using polyester suture tape and knotless anchors and concluded that fixation should be conducted in 60°–90° of flexion to most closely restore the PF-joint contact pressure. Thus, we consistently fixed the graft at 60° of knee joint flexion in all of the conducted procedures with an applied low tension of 2 N as previously recommended [14].

To the best of our knowledge, this is only the second study that compared the results of MPFL-R using nonresorbable sutures with the results of established autograft MPFL-R, and it is the first study to investigate patient-reported outcome measures after S-MPFL-R in combination with the correction of bony pathoanatomy in comparison with the outcomes in a matched control group treated with identical primary stabilizing procedures but using QT-MPFL-R. However, the results of this study must be interpreted under the consideration of several limitations: (1) Although statistical analysis showed that the number of patients per group provides sufficient power, the overall number of included patients can be regarded as small. Against this background, the results of this study should be considered preliminary and simply indicate the feasibility of this technique. (2) Between the groups, the follow-up period was significantly different (24.6 ± 1.3 vs. 35.8 ± 9.9 months). This compromise had to be made in favor of the best possible homogeneity between the groups regarding the demographic data, the characteristics and severity of the anatomical risk factor profile and their operative correction, which we considered most important. (3) The follow-up period was limited to a minimum of 2 years postoperatively in the S-MPFL-R group. This does not allow us to fully evaluate the long-term application potential of this novel technique, especially when considering that synthetic materials cannot be replaced by autologous tissue. However, no prolonged swelling, tenderness, incompatibility or foreign body reactions in patients treated with the proposed reconstruction were observed. (4) It is important to note that this study evaluated only patient-reported outcomes, and no clinically objective measures, such as functional testing, were conducted. Finally, the biomechanical properties of this particular reconstruction remain to be determined in future studies.

## Conclusion

This study demonstrates that nonresorbable sutures can serve as a viable option for MPFL-R, yielding comparable outcomes compared to quadriceps tendon autograft reconstruction when performed concomitantly with the correction of anatomic risk factors for LPI. This option reduces the need for autologous tendon harvesting or the use of allografts for MPFL-R.

## Data Availability

Raw data can be made available upon request.
